# Sensorimotor Responses in Post-Stroke Hemiplegic Patients Modulated by Acupuncture at Yanglingquan (GB34): A fMRI Study Using Intersubject Functional Correlation (ISFC) Analysis

**DOI:** 10.3389/fneur.2022.900520

**Published:** 2022-06-06

**Authors:** Yue Wang, Liping Wang, Yahui Wang, Mengxin Lu, Lingling Xu, Ruoyi Liu, Jingpei Wei, Jifeng Wan, Hua Zhang, Yihuai Zou

**Affiliations:** Department of Neurology, Dongzhimen Hospital, Beijing University of Chinese Medicine, Beijing, China

**Keywords:** sensorimotor cortex, Yanglingquan (GB34), acupuncture, hemiplegia, motor dysfunction, functional MRI, intersubject functional correlation, ischemic stroke

## Abstract

Motor dysfunction is common in patients with stroke. Acupuncture has become an acceptable alternative method for stroke rehabilitation. Previous studies have shown various functional connectivity changes activated by acupuncture. We introduced intersubject correlation (ISC) and intersubject functional correlation (ISFC) analyses into the functional magnetic resonance imaging (fMRI) for ischemic stroke to seek a common activation and suppression pattern triggered by acupuncture. In this study, 63 ischemic stroke patients with motor dysfunction and 42 normal controls were analyzed. Three functional scans were conducted during the resting state, motor task, and acupuncture at Yanglingquan (GB34) task. Twenty-two sensory, motor, and movement-imagination cortices in the bilateral hemispheres were selected as the region of interest (ROI). We performed ISC and ISFC analyses among these ROIs in three fMRI runs on patients and controls. Subgroup analyses by course or severity were also conducted. The results showed that acupuncture at GB34 triggered ISFC among upper limb motor, upper limb/hand/face, lower limb, tongue/larynx sensory, and movement imagination regions in the patient group. Subgroup ISC and ISFC analyses showed that patients tended to have increasing responses in the early stage of stroke (within 1 month) and decreasing responses afterward (1–3 months). Patients with mild clinical functional damage (NIHSS 2–4) tended to generate more responses *via* acupuncture than those with moderate damage (NIHSS 5–15). Our findings may help understand the clinical effects and modulatory features of acupuncture based on the group-level post-stroke neuroplasticity.

## Introduction

Stroke, the third leading cause of death and disability, has been causing a serious disease burden worldwide (1). Based on current stroke trends, the World Stroke Organization (WSO) has estimated that there will be about 200 million stroke survivors in 2050 (2). The probability of post-stroke motor dysfunction is about 50–85%. Thus, functional recovery and rehabilitation, which focus on neuronal plasticity and brain network recovery after stroke, have been the key transnational research topics (3). The current knowledge of post-stroke neuroplasticity is mainly based on invasive methods (4), which cannot be performed in clinical trials. On the contrary, non-invasive functional magnetic resonance imaging (fMRI) has been widely applied in the field of post-stroke neural plasticity with outstanding features of high spatial and temporal resolution (5, 6).

Post-stroke rehabilitation can improve motor function and regulate neuroplasticity (7, 8). Noteworthy, upper extremity function is particularly important for functional training, as it can determine whether an individual can return to his formal professional and personal life (9). Thumb-index-finger opposition is routine training for post-stroke patients with upper limb dysfunction. Both active and passive hand practices are beneficial in improving motor function (10, 11). fMRI-based research has found that 1-Hz thumb-index-finger opposition mainly activated the primary and secondary motor regions, which extended to the posterior central gyrus and anterior central sulcus (12).

Acupuncture, a traditional Chinese therapy, has a gradually increasing geographical coverage and application rate (13). The NIH Consensus Conference (14) and publications (15, 16) have demonstrated that acupuncture can improve the neurological motor deficit symptoms and become an acceptable alternative method for stroke rehabilitation. Recent research has shown that acupuncture was involved in regulating neural plasticity in patients with stroke, both functionally and structurally (17, 18). Yanglingquan (GB34), the He-Sea point of the gall bladder meridian (Foot-Shaoyang), the confluence point of tendons, is one of the main points in post-stroke acupuncture approaches, such as Wang's twelve-needling at hands and feet method and Xingnao Kaiqiao needling method (19). GB34 has been the most commonly used acupoint to study the mechanism of acupuncture stimulation in stroke based on fMRI (20). Previous studies have proposed that acupuncture at GB34 could enhance the functional connectivity (FC) between bilateral M1 cortices and increase motor-cognition connectivity (21, 22). The conventional analysis has been displayed by calculating the correlations of time series in the target regions of every single subject. In addition to the real signal induced by stimulation, FC also contains information that cannot be well-separated, such as intrinsic neural signal and non-neural noise signal (23). Therefore, capturing simpler and more accurate neural activities triggered by acupuncture among different subjects is a topic requiring attention.

The intersubject correlation (ISC) analysis provides a data-driven alternative for seeking the activity caused by complex stimuli. Based on the logic of ISC, intersubject functional correlation (ISFC) represents a new analytical method for revealing the functional connectivity of stimuli in various brain regions across subjects (24). Similar to the functional connectivity analysis, ISFC analysis targets the correlation between two response time series; however, rather than calculating the correlation between two regions within a subject, ISFC analysis calculates the same two regions across subjects. A comparative study has revealed that the data-driven ISC analysis found the same foci as the model-based general linear model (GLM) analysis and was applicable in situations where GLM was not suitable (25). Currently, this method has been widely used in studies of visual and auditory stimuli (26–28). We innovatively introduced this method into our study to further explore the common neural activities of focal brain regions in patients with post-ischemic stroke hemiplegia.

With the development of image technology and the sharing of neuroimaging information, researchers have proposed a new brain atlas, which divides the brain into 246 regions based on functional connectivity information. Unlike previous atlases that only represented specific structures and lacked fine-grained parcellations, the Brainnetome (BN) atlas has more fine-grained functional brain subregions and detailed functional connection patterns, which can help more accurately describe the locations of the activation or connectivity in the brain (29). In this study, we selected 22 sensory, motor, and movement-imagination-related cortices in bilateral anterior central gyri, posterior central gyri, and their vicinity as the regions of interest (ROI) based on the BN atlas to focus on the deficit-related rehabilitation mechanisms on hemiplegic patients by acupuncture.

This study aimed to explore the cross-subject effects of acupuncture at GB34 on functional sensorimotor activities among ischemic stroke patients with hemiplegia. Healthy subjects were recruited as normal controls. A resting-state scan was used as a background observation. Thumb-index-finger opposition was used as a reference motor task. Activation and suppression correlation responses among 22 ROIs chosen from the BN atlas were calculated and presented by applying the ISFC analysis.

## Materials and Methods

### Participants

**Figure 1:**
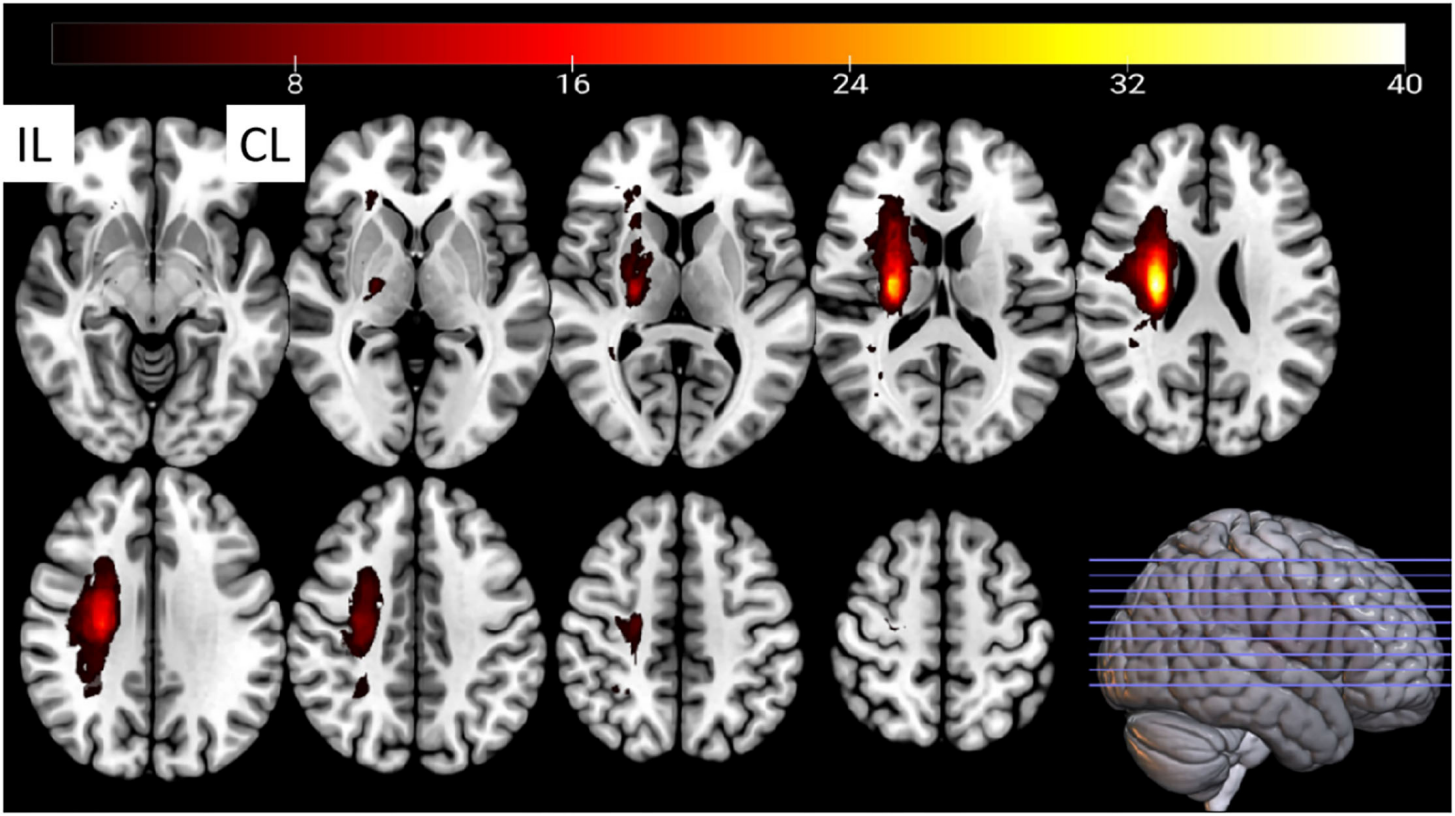
The stroke patients' lesion-incidence map. CL, contralesional; IL, ipsilesional. Fifteen lesions from the left hemisphere were flipped to the right hemisphere.

This study included 63 right-handed post-stroke inpatient subjects with hemiplegia from a single center (Dongzhimen Hospital, Beijing University of Chinese Medicine). A total of 42 age- and sex-matched healthy subjects were recruited as normal controls. All participants signed informed consent before inclusion, which explained the whole study design, including the purpose and procedures of this study, the benefits and risks of being involved, and the rights of self-data acquirement and being able to quit without conditions. Participants received a signed copy of the informed consent. Basic information (age, sex, and vital signs), National Institute of Health stroke scale (NIHSS) score, and functional and structural MRI data were collected on the same day. The subjects' demographic features and patients' lesion incidence map are shown in Table 1 and Figure 1, respectively. All patients included in this study had mild to moderate (NIHSS of 0–15) neural dysfunction.

**Table 1:** Demographic features and clinical information of patients with stroke and normal controls.

	**Number**	**Sex (male/female)**	**Age**	**Stroke time (day)**	**NIHSS score**	**Number of patients with right hemisphere lesion**	**Number of patients with left hemisphere lesion**
Patient Group	63	43 / 20	59.56 ± 9.82	27.22 ± 17.53	3.81 ± 3.19	48	15
Control Group	42	22 / 20	56.64 ± 5.17	-	-	-	-

*No significant distribution of age difference (two-sample t-test, p > 0.05) or sex difference (χ^2^-test, p > 0.05) detected. Enumeration data are presented as mean ± standard deviation*.

The included patients met the following criteria: (1) patients with ischemic stroke who conform to the diagnostic criteria (30) and whose course of the disease is within 3 months; (2) right-handed patients; (3) patients aged 40–75 years; (4) patients presenting first-ever post-stroke motor dysfunction in the upper or lower extremities; (5) patients in whom the infarction was located in the basal ganglia and/or the corona radiate region, side-unlimited, but not caused by embolism; (6) patients without other central nervous system diseases (e.g., epilepsy, Parkinson's disease, Alzheimer's disease, and vascular dementia), psychiatric disorders (e.g., depression and schizophrenia), or any other serious primary diseases (e.g., tumors, organ failure, atrial fibrillation, and thrombotic diseases); (7) patients without unremovable metallic implants or contraindication of magnetic resonance examination.

The included healthy controls met the following criteria: (1) right-handed people; (2) people aged 40–75 years; (3) people proved to be healthy by a medical examination (without organic or significant functional diseases); (4) people without a family history of mental or neural system inheritance; (5) people without experience of joining research similar to this experiment; (6) people without physical abnormalities (e.g., cold, headache, and cough) during the trial; (7) people not taking any excitatory medications within the past 2 months; and (8) people without unremovable metallic implants or contraindication of magnetic resonance examination.

### Task Design

**Figure 2:**
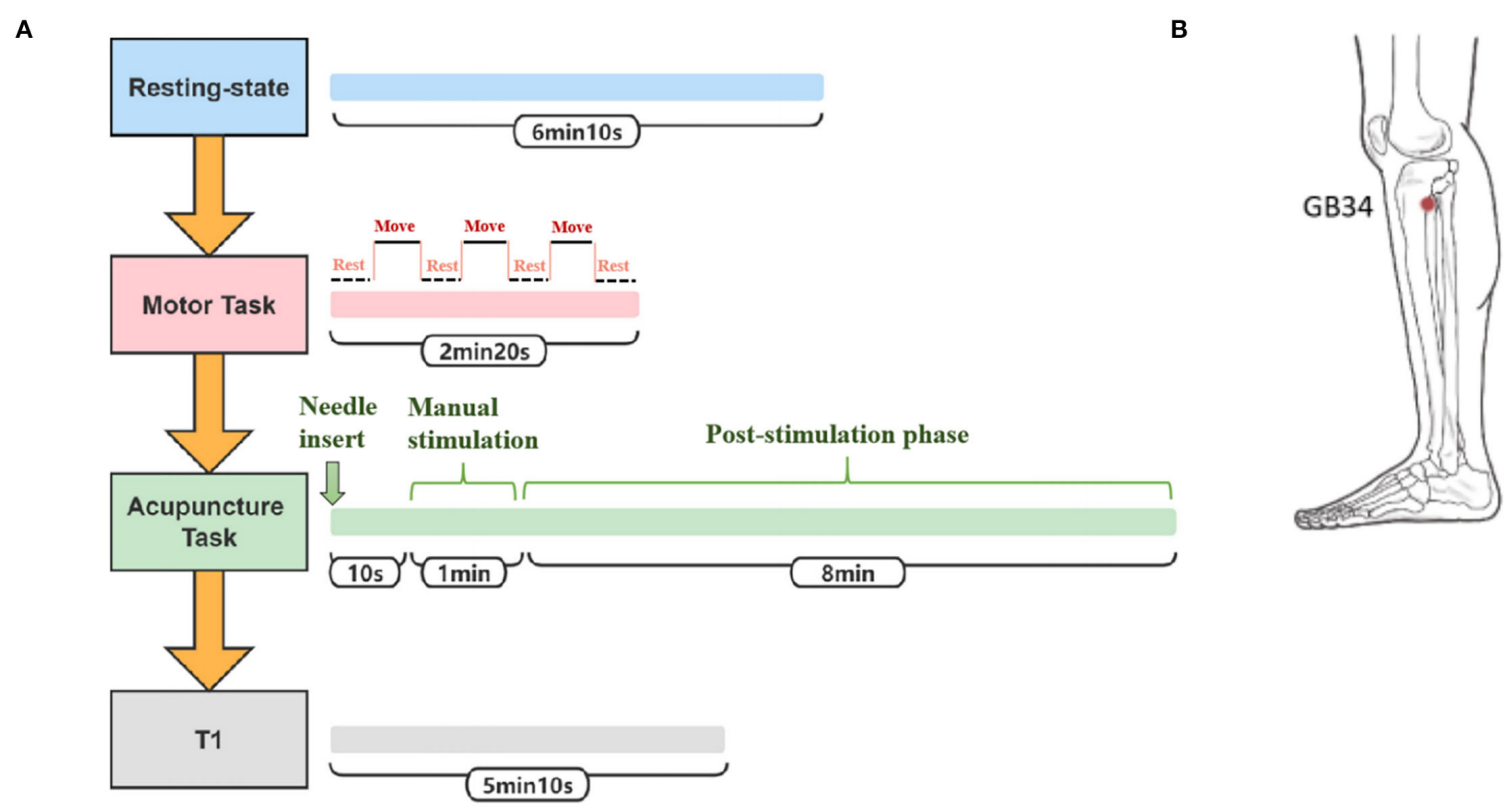
Study design. **(A)** The scanning protocol. **(B)** The location of Yanglingquan (GB34).

The participants were told to close their eyes without engaging in any mental task. According to participants' reports after the scanning, they were affirmed to keep awake during the whole scanning procedure. Three fMRI runs were conducted. The first run was in a resting state for 6 min and 10 s. The second run occurred during the motor task with passive thumb-index-finger opposition on the affected side in patients or the left side in normal controls. The task was performed as a “rest-move-rest-move-rest-move-rest” pattern by two researchers. Each time slice lasted for 20 s for a total of 2 min and 20 s. The “move” period allowed the participants' thumb and index fingertips touching-and-parting at a frequency of 1 Hz (31). The “rest” period had no intervention, only relaxation. The third run occurred during the acupuncture operation on the affected side in patients or the left side in normal controls. The needles were disposable sterile silver needles (specification parameter: ϕ0.40 × 40 mm) purchased from Beijing Zhongyantaihe Medical Instrument Co., LTD., and manufactured by Suzhou Shenlong Medical Instrument Co., LTD. Acupoint selection was performed according to the National standard GB/T 12346-2006 Name and Location of Acupoints. Yanglingquan (GB34) is located on the outside of the lower leg, in the middle of the concavity of the anterior and inferior parts of the fibula head. After routine skin disinfection, the needle was vertically inserted for 1.5–2 Cun (about 25–40 mm depending on the height and weight of a participant) at GB34. There was a 10-s post-onset phase of the resting state with the needle inserted, followed by a 1-min manual stimulation phase by using the mild reinforcing–reducing method at a frequency of 1 Hz. Then, an 8-min post-stimulation phase occurred with the needle remaining inside the leg. The needle was removed and disposed of after the acupuncture scanning. In the end, the T1-weighted structural image was acquired. The scanning protocol and position of GB34 are shown in Figures 2A,B, respectively. At the end of the MRI scanning procedure, NIHSS scores of patients with ischemic stroke were evaluated by an experienced neurologist.

### fMRI Parameters

All scans were performed by a 3.0 Tesla scanner (Siemens, Verio, Germany) in Dongzhimen Hospital. The scanning parameters were as follows: fMRI was applied with echo-planar imaging (EPI) sequence with a repetition time (TR) of 2,000 ms; echo time (TE) of 30 ms; matrix of 64 × 64; field of view (Fov) of 225 × 225 mm; slice thickness of 3.5 mm; a gap of 0.7 mm; phase encode direction of A >> P; flip angle of 90°; and fat suppr of fat sat. The three-dimensional structure imaging scan of the whole brain was scanned using the following T1W1 sequence: TR of 1,900 ms; TE of 2.53 ms; Fov of 250 × 250 mm; matrix of 256 × 256; and slice thickness of 1.0 mm.

### Data Processing

For each of the 3 BOLD runs per subject, the following preprocessing was performed (32). First, a reference volume and its skull-stripped version were generated. BOLD runs were slice-time corrected to 0.971 s using 3dTshift from AFNI (RRID:SCR_005927). Head-motion parameters with respect to the BOLD reference were estimated using mcflirt (FSL 5.0.11). The BOLD time series were resampled onto their original native space by applying the transforms to correct for head motion. The BOLD reference was then co-registered to the T1w reference using bbregister (FreeSurfer) with 6 degrees of freedom. Framewise displacement (FD), DVARS, and 3 region-wise global signals within the cerebrospinal fluid, the white matter, and the whole-brain masks were calculated. Additionally, a set of physiological regressors was extracted to allow component-based noise correction. Finally, these masks were resampled into BOLD space and binarized by thresholding at 0.99. The BOLD time series were resampled onto the fsnative. Automatic removal of motion artifacts using an independent component analysis (ICA-AROMA) was performed on the preprocessed BOLD on the Montreal Neurological Institute (MNI) space-time series after the removal of non-steady state volumes and spatial smoothing with an isotropic Gaussian kernel of 6 mm FWHM (full-width half-maximum). Imaging data were filtered (bandpass, 0.01–0.1 Hz; Supplementary Material 1).

### Statistical Analysis

**Figure 3:**
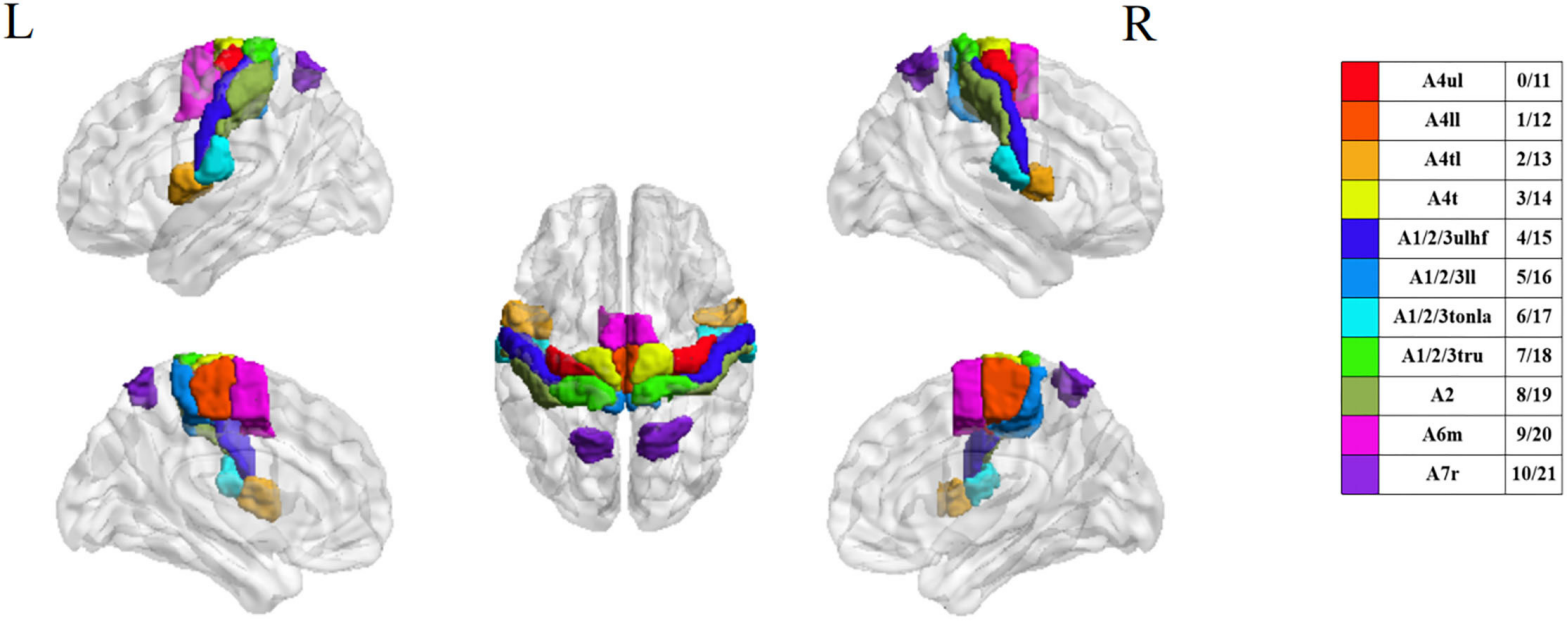
ROI distribution.

The BN atlas was downloaded from https://atlas.brainnetome.org/index.html and used to define the location of the ROIs of all three fMRI datasets. The average time series of the voxel in each ROI were calculated according to the spatial coordinates. For the 15 patients with left-sided lesions, we exchanged the data symmetrically between the two hemispheres. The chosen ROIs were motor, sensory, and movement-imagination cortices (Table 2 and Figure 3). Also, the exchange of the data would not concern the specific dysfunction of the dominant hemisphere, such as aphasia. By this step, all 63 patients with right hemisphere lesions received affected-side passive finger movement and GB34 acupuncture operation.

**Table 2:** Information of 22 ROIs based on Brainnetome Atlas.

**No**.	**Name**	**Hemisphere**	**Description**	**Location**
0	A4ul_L	Left	Upper limb region	Precentral gyrus
1	A4ll_L	Left	Lower limb region	Paracentral lobe
2	A4tl_L	Left	Tongue/larynx region	Precentral gyrus
3	A4t_L	Left	Trunk region	Precentral gyrus
4	A1/2/3ulhf_L	Left	Upper limb/hand/face region	Postcentral gyrus
5	A1/2/3ll_L	Left	Lower limb region	Paracentral lobe
6	A1/2/3tonla_L	Left	Tongue/larynx region	Postcentral gyrus
7	A1/2/3tru_L	Left	Trunk region	Postcentral gyrus
8	A2_L	Left	S2 sensory	Postcentral gyrus
9	A6m_L	Left	Medial area 6	Superior frontal gyrus
10	A7r_L	Left	Rostral area 7	Superior parietal lobe
11	A4ul_R	Right	Upper limb region	Precentral gyrus
12	A4ll_R	Right	Lower limb region	Paracentral lobe
13	A4tl_R	Right	Tongue/larynx region	Precentral gyrus
14	A4t_R	Right	Trunk region	Precentral gyrus
15	A1/2/3ulhf_R	Right	Upper limb/hand/face region	Postcentral gyrus
16	A1/2/3ll_R	Right	Lower limb region	Paracentral lobe
17	A1/2/3tonla_R	Right	Tongue/larynx region	Postcentral gyrus
18	A1/2/3tru_R	Right	Trunk region	Postcentral gyrus
19	A2_R	Right	S2 sensory	Postcentral gyrus
20	A6m_R	Right	Medial area 6	Superior frontal gyrus
21	A7r_R	Right	Rostral area 7	Superior parietal lobe

Then ROIs time series were extracted from the preprocessed fMRI data. ISC and ISFC for each functional run were calculated and analyzed across the subjects from each group by using Brain Imaging Analysis Kit (BrainIAK, https://brainiak.org) implemented in Python rather than computing the correlations across the ROIs within one subject. We used the isc function in BrainIAK with a leave-one-out approach comprising BOLD time series for one ROI across multiple subjects and returns ISC values for ROIs for each subject. For the ISFC analysis, we supplied BOLD time-series data for 22 ROIs across the subjects to the isfc function. By using the leave-one-out approach, we plotted the ROI-by-ROI ISFC matrix for each subject. Then, we used the bootstrap_isc function (applying a bootstrap hypothesis test) to perform group-level statistical tests on the subject-level ISC/ISFC values. We computed confidence intervals as 95%, random state as 1,000, and resampling iterations as 1,000 in this step and obtained group-level ROI-to-ROI *p*-values and mean ISC/ISFC values. For the group comparison, we conducted the Monte Carlo approximate permutation tests between the two groups (e.g., patients with stroke vs. normal controls, motor task vs. acupuncture task). False discovery rate (FDR) correction was used for multiple tests across the ROIs.

## Results

### Resting-State ISC and ISFC

**Figure 4:**
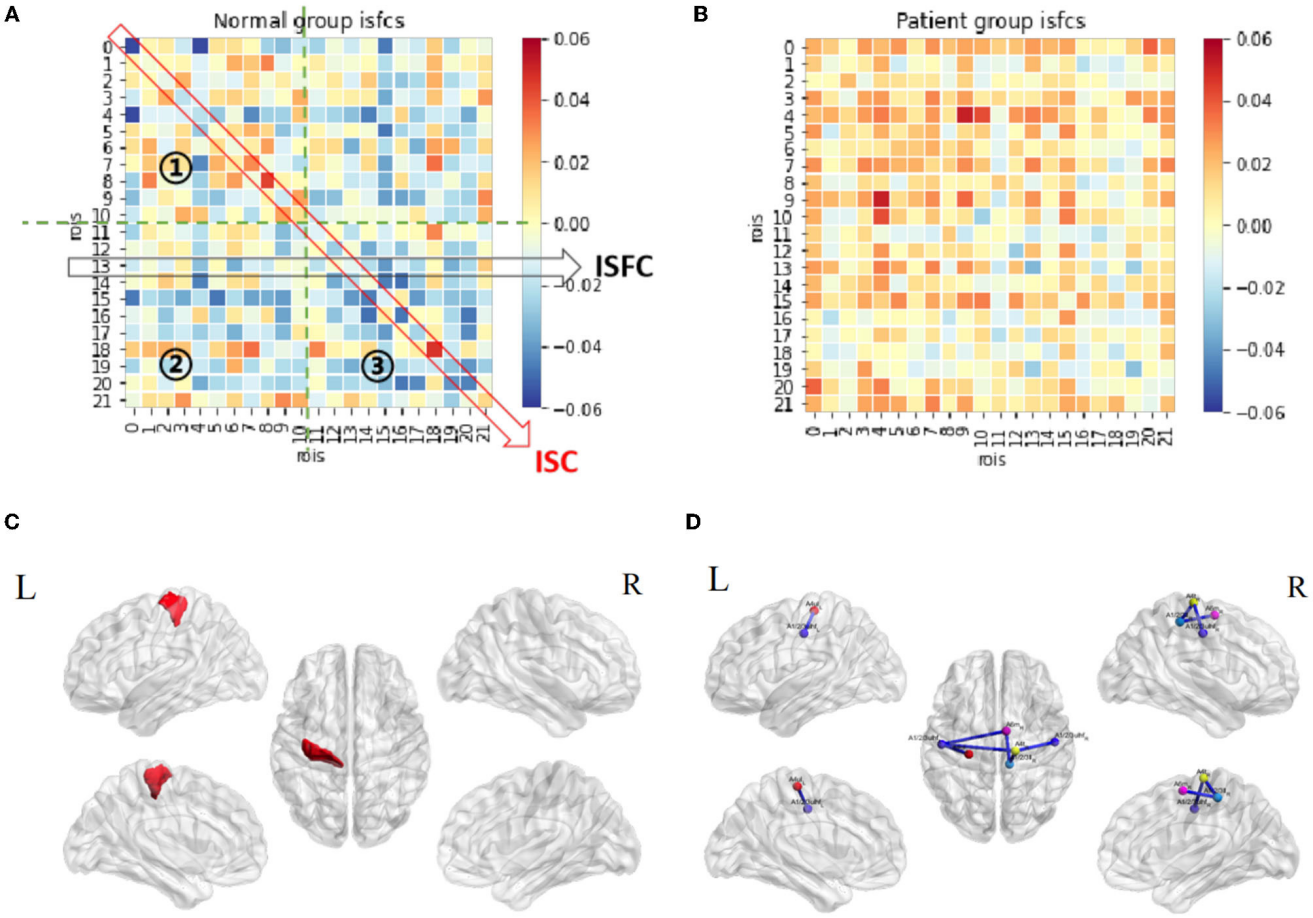
Resting-state ISC and ISFC. **(A)** The ISFC matrix of 22 ROIs during resting state in the control group. The off-diagonal values represent intersubject functional correlations between ROIs, while the diagonal values represent intersubject correlation within each ROI. ①, ②, and ③ represent the area of contralesional hemisphere, between hemispheres, and ipsilesional hemisphere (the other matrices in this article follow this law). **(B)** ISFC matrix of 22 ROIs during resting state in the patient group. **(C)** Significant ROI at resting-state in the control group (*p* < 0.05, FDR-corrected). **(D)** Significant negative ISFCs (lines in blue) between ROIs in the control group (*p* < 0.05, FDR-corrected).

The patterns of ISC and ISFC across the ROIs in the patient and control groups were first observed. We generated the functional correlation matrix across all 22 ROIs (Figure 4A). The off-diagonal values in the matrix were time-series correlations between the ROIs. The diagonal values were correlations between subjects of one particular ROI (ISC). In the control group, the correlations varied and were distributed evenly, having more negative values than positive values. Statistical analysis of ISC showed that region A4ul_L was spontaneously activated in the control group (*p* < 0.05, FDR-corrected; Figure 4C). ISFC analysis showed several negative correlations between the ROIs in the control group (*p* < 0.05, FDR-corrected; Figure 4D): A1/2/3ulhf_L and A4ul_L, A1/2/3ulhf_L and A4t_R, A1/2/3ulhf_L and A6m_R, A1/2/3ulhf_R and A4t_R, A1/2/3ll_R and A4t_R, A1/2/3ll_R and A6m_R. These pairs were between the upper body sensory cortex, trunk motor and sensory cortex, and movement–imagination cortex, which indicated that there were only a few spontaneous negative connectivities in normal controls. In contrast, the ISFC matrix in the patient group (Figure 4B) had a relatively consistent pattern of positive values. Notably, mild positive values were universal between the ROIs within the contralesional (left) hemisphere. Additionally, negative correlations were a handful and gentle across the matrix. There were no significant ROIs or ROI pairs found in the patient group by statistical analysis (*p* > 0.05, FDR-corrected). These findings proved that ISFC analysis could filter out individual and stimulus-unrelated neural signals. Furthermore, patients had less shared response between the regions than controls in the resting state.

### Motor Task-Stimulated ISC and ISFC

**Figure 5:**
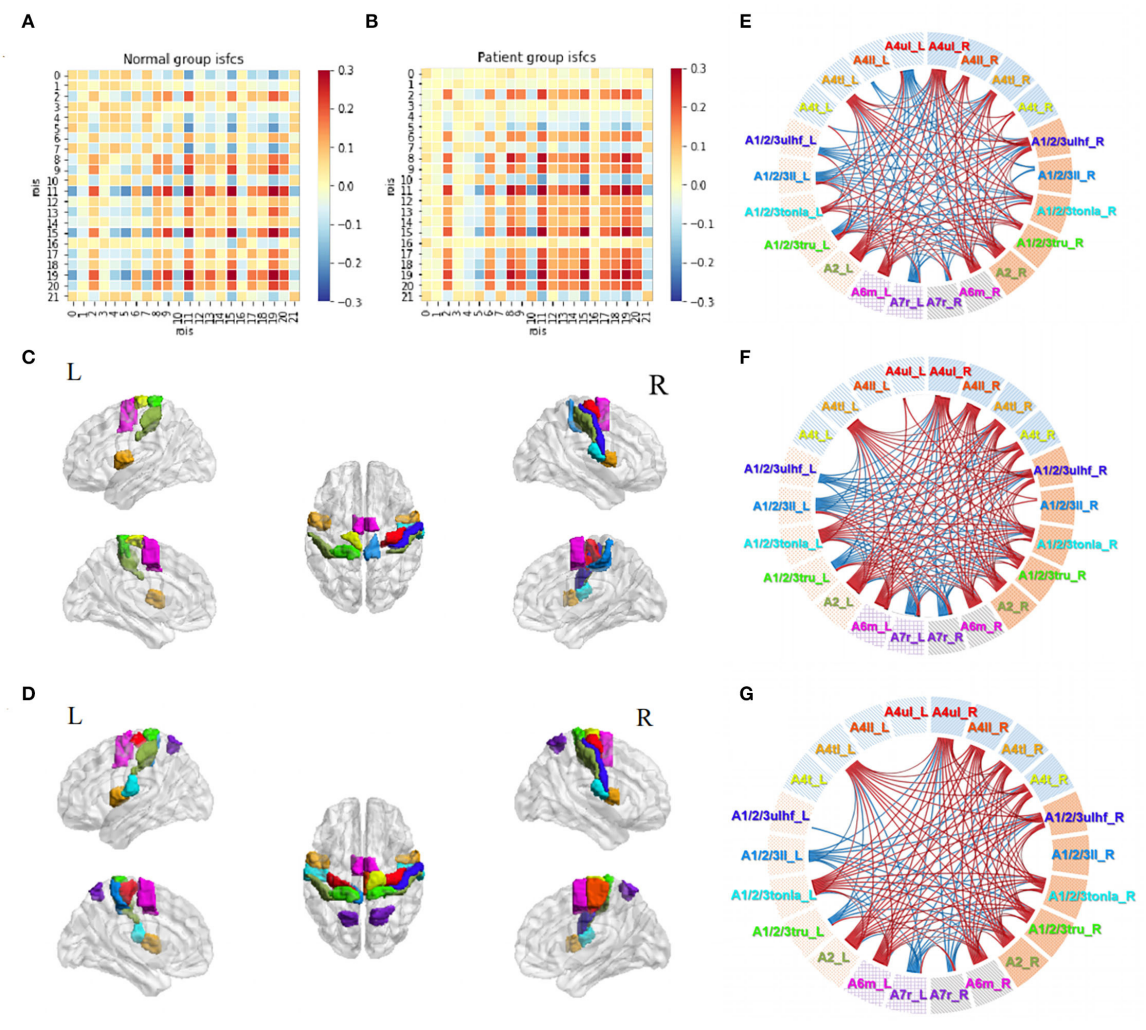
Motor task-stimulated ISC and ISFC. **(A)** ISFC matrix of 22 ROIs during motor task in the control group. **(B)** ISFC matrix of 22 ROIs during motor task in the patient group. **(C)** Significantly stimulated ROIs in the control group (*p* < 0.01, FDR-corrected). **(D)** Significantly stimulated ROIs in the patient group (*p* < 0.01, FDR-corrected). **(E)** Significant positive (lines in red) and negative (lines in blue) ISFCs between ROIs in the control group (*p* < 0.01, FDR-corrected). **(F)** Significant ISFCs between ROIs in the patient group (*p* < 0.01, FDR-corrected). **(G)** Significant ISFCs between ROIs in the patient group (*p* < 0.001, FDR-corrected).

Functional correlation matrices based on the motor task were observed in both groups (Figures 5A,B). The motor task triggered a large scope of responses in both groups. Patients with stroke and normal controls had high-level positive correlations between the ROIs in the ipsilesional (right) hemisphere, especially between A4ul_R, A1/2/3ulhf_R, A2_R, and A6m_R, which were hand-related sensorimotor and imagination cortices. Patients tended to have higher positive correlations among the ROIs in the ipsilesional hemisphere than normal controls except for regions A1/2/3ll_R and A7r_R, which had zero or negative values with others. Within the contralesional hemisphere, both groups had a similar pattern as in the resting state, having lower values among the ROIs. For the inter-hemisphere correlations, the two groups had similar patterns.

Statistical analysis demonstrated a substantial amount of significant ISCs and ISFCs (*p* < 0.01, FDR-corrected). In the ISC analysis, patients had a wider activation range than normal controls, with activated bilateral upper limb motor regions (A4ul_R, A4ul_L; Figures 5C,D). In ISFC analysis (Figures 5E,F), the findings were (1) most ROIs time series positively correlated with others within the ipsilesional hemisphere in both groups; (2) there was an equal amount of negative and positive correlations between the hemispheres in both groups. In contrast, in the control group, regions A4ul_L and A4ll_L tended to have many negative correlations with others, which were not seen in the patient group. Our study of ISCs and ISFCs during the motor task demonstrated that one-side passive thumb-index-finger opposition movement could activate shared positive correlations of sensorimotor cortices within the contralateral hemisphere and several negative correlations between the bilateral hemispheres. On comparing the groups, patients had more significant pairs within the contralateral side and more positive and less negative pairs between the bilateral upper/lower body ROIs. The differences we observed were in line with the common knowledge that disturbances after stroke not only occur in the vicinity of the lesion but also between remote cortical areas in the affected and unaffected hemispheres (33). Additionally, when we narrowed down the statistical *p*-value to *p* < 0.001 (FDR-corrected), the patient group retained almost entirely significant ISFCs from the level of *p* < 0.01 (Figure 5G), while the control group showed no significant values. The cortical functional connections under pathological conditions seemed to be more accordant.

### Acupuncture Task-Stimulated ISC and ISFC

**Figure 6:**
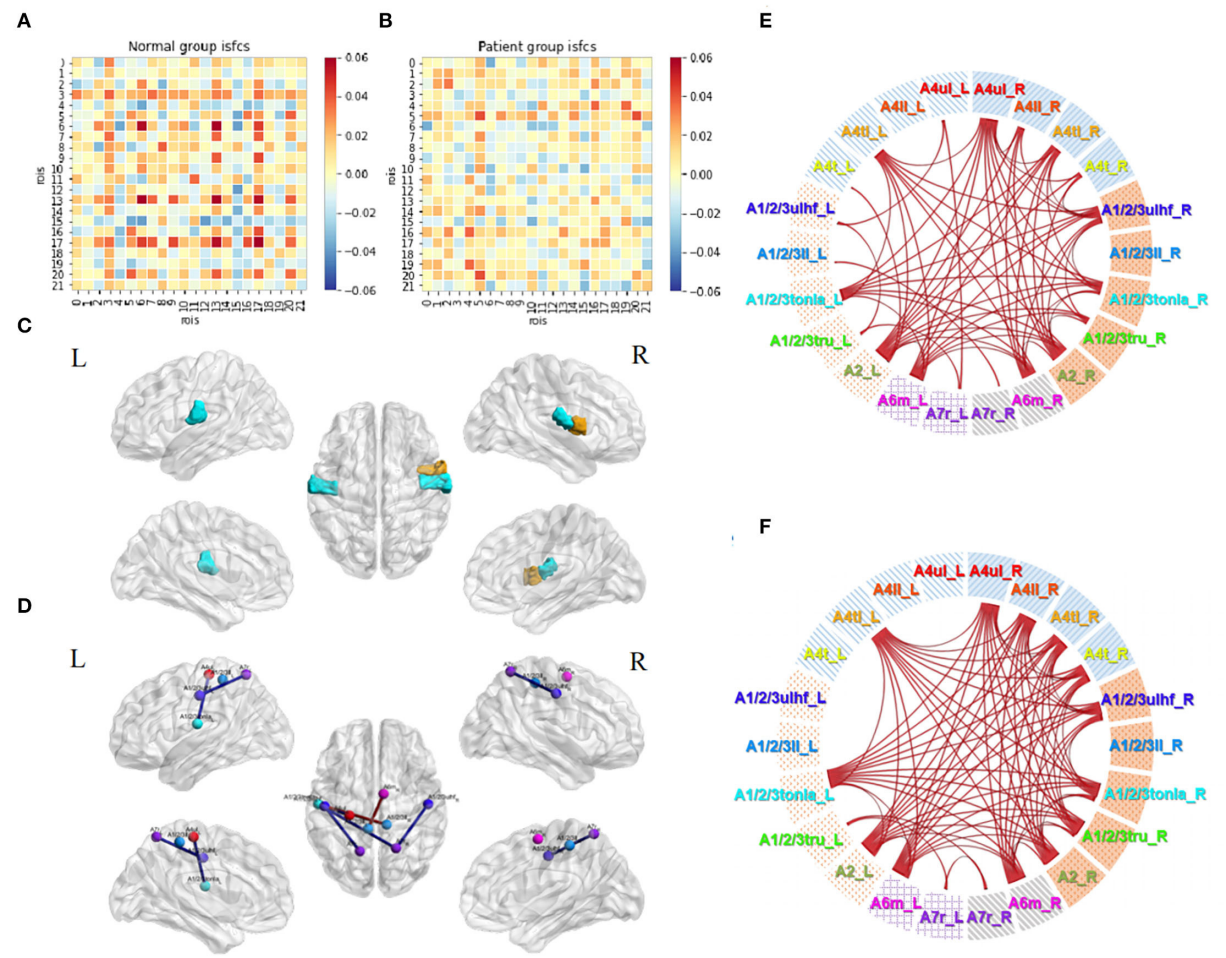
Acupuncture task-stimulated ISC and ISFC. **(A)** ISFC matrix of 22 ROIs during acupuncture task in the control group. **(B)** ISFC matrix of 22 ROIs during acupuncture task in the patient group. **(C)** Significant ROIs in the control group during acupuncture (*p* < 0.05, FDR-corrected). **(D)** Significant positive (lines in red) and negative (line in blue) ISFCs during acupuncture in the patient group (*p* < 0.05, FDR-corrected). **(E)** Significant ISFCs between motor task vs. acupuncture task in the control group (*p* < 0.01, FDR-corrected). **(F)** Significant ISFCs between motor task vs. acupuncture task in the patient group (*p* < 0.01, FDR-corrected).

The acupuncture task demonstrated unique patterns of correlation distributions (Figures 6A,B), different from either the resting state or the motor task in both groups. In the control group, unlike in the motor task, the positive and negative values were distributed evenly within and between the hemispheres. Unlike in the resting state, more positive values were observed during the acupuncture procedure. Patients' ISFCs showed even distribution as well, differing from either the resting state (positive values within the unaffected hemisphere) or motor task (positive values within the affected hemisphere). The matrix of patients was visibly weaker than normal controls, which indicated that patients had slighter connectivity changes.

Statistical analysis in the control group showed that regions A1/2/3tonIa_L, A4tl_R, and A1/2/3tonIa_R were activated by acupuncture (*p* < 0.05, FDR-corrected; Figure 6C). Surprisingly, no significant ROI pairs appeared (*p* > 0.05, FDR-corrected). Although subjects had various responses and their tongue/larynx sensorimotor areas were activated during acupuncture, no synchronous functional correlation between the ROIs across all subjects existed. The significantly increased functionally connected pairs stimulated by acupuncture in patients were A1/2/3ulhf_L and A1/2/3ll_R, A1/2/3ll_L and A6m_R, and decreased pairs were A4ul_L and A1/2/3tonIa_L, A1/2/3ulhf_L and A7r_L, A7r_R and A1/2/3tonIa_L, A7r_R and A1/2/3ulhf_R (Figure 6D; *p* < 0.05, FDR-corrected). The regulation mainly occurred within the contralesional hemisphere and between the hemispheres.

By putting the significant maps during the motor and acupuncture tasks in patients with stroke side by side, we noticed a common trend of opposite activation of bilateral A7r with sensory regions. While the movement stimulus had a strong and targeted stimulation on the ROIs we obtained, immediate acupuncture stimulation at GB34 presented a relatively less, but comprehensive response. Comparison between the two tasks in both groups showed consistent higher correlations among ROI pairs (Figures 6E,F; *p* < 0.01, FDR-corrected). Thus, the motor task activated significantly more abundant connections than the acupuncture stimulus, especially in the affected hemisphere, whereas the negative correlations triggered by the two tasks were not significantly different.

### Subgroup Analyses of Acupuncture Tasks in Patients

We performed a subgroup analysis based on the time course of stroke and neurological damage to further explore the cross-subject bilateral sensorimotor connections in patients with stroke during acupuncture. Subgroup characteristics are reported in Table 3.

**Table 3:** Demographic features and clinical data of subgroup patients.

**Subgroup**	**Number**	**Sex (male/female)**	**Age (year)**	**Course (day)**	**NIHSS**
C1	16	13 / 3	57.44 ± 11.10	8.81 ± 3.17	3.44 ± 2.94
C2	24	14 / 10	59.21 ± 9.99	20.54 ± 4.16	3.38 ± 3.57
C3	23	16 / 7	61.39 ± 8.76	47 ± 11.68	4.52 ± 2.92
Nih1	18	12 / 6	55.56 ± 6.56	22.28 ± 16.68	0.44 ± 0.51
Nih2	21	17 / 4	61.48 ± 9.11	24.38 ± 14.18	2.9 ± 0.77
Nih3	24	14 / 10	60.88 ± 11.76	33.42 ± 19.52	7.13 ± 2.37

*Enumeration data are presented as mean ± standard deviation. No significant different NIHSS scores were detected among subgroup C (ANOVA, p > 0.05). No significant different course was detected among subgroup Nih (ANOVA, p > 0.05)*.

**Figure 7:**
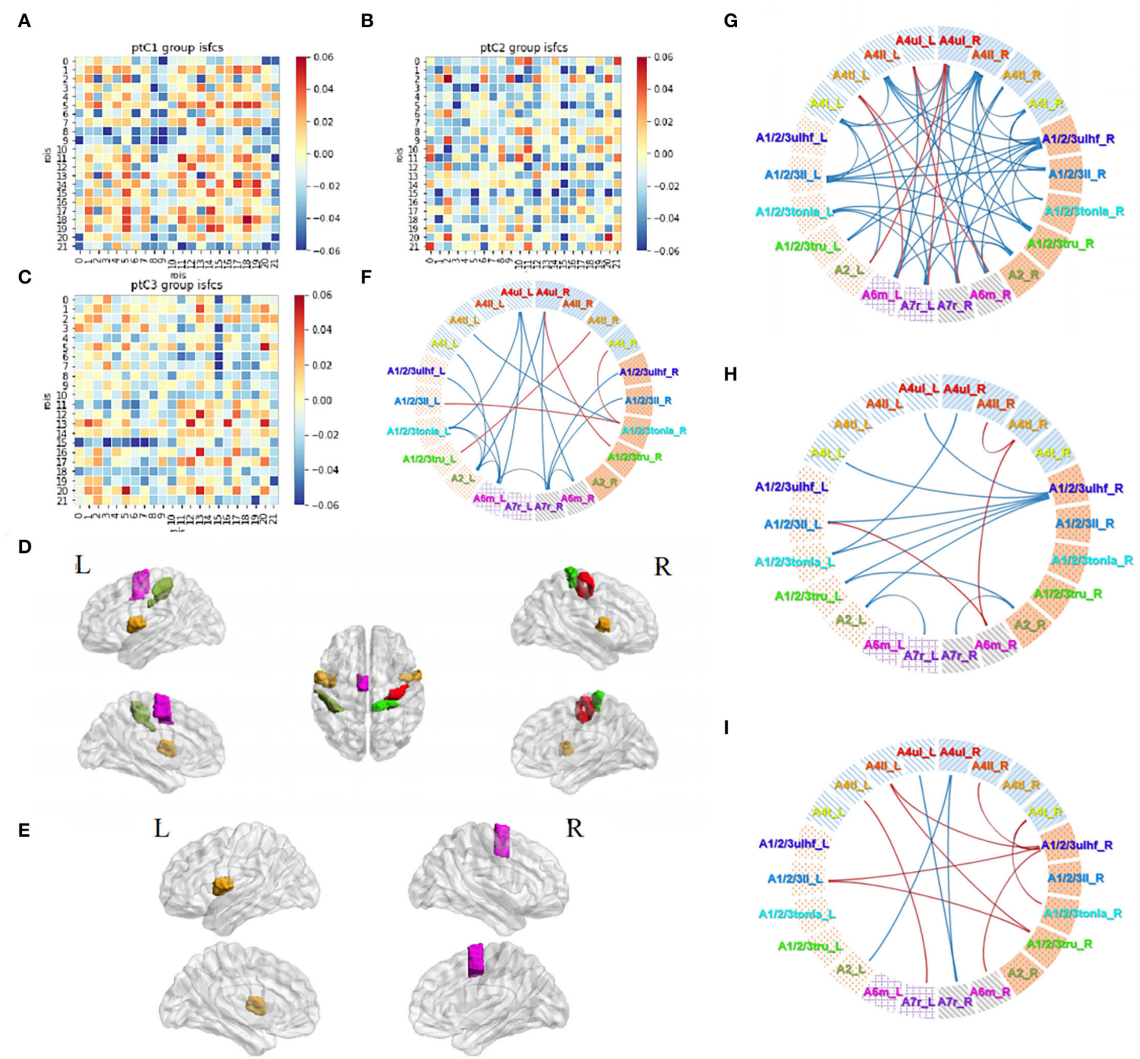
Acupuncture task-stimulated ISC and ISFC in subgroup Cs. **(A–C)** ISFC matrix of 22 ROIs in C1, C2, C3. **(D,E)** Significantly stimulated ROIs in C1, C2 (*p* < 0.05, FDR-corrected). **(F–H)** Significant ISFCs between ROIs in C1, C2, C3 group (*p* < 0.05, FDR-corrected). **(I)** Significant ISFCs between C1 vs. C2 (*p* < 0.05, FDR-corrected).

Firstly, 63 patients were divided into 3 groups based on the course: group C1, within 0.5 month (16 subjects); group C2, 0.5–1 month (24 subjects); and group C3, 1–3 months (23 subjects). Correlations in three subgroups were stronger than themselves when calculated as a whole group. The connection degrees showed a trend of strong to weak distributions from group C1 to group C3 (Figures 7A–C). Significant activities in ROIs and ROI pairs were different across the groups (*p* < 0.05, FDR-corrected). Specifically, C1 group patients had their regions A4ul_R (ipsilesional upper limb motor area), A4tl_R, and A4tl_L (bilateral tongue/larynx motor area), A2_L, A6m_L, and A1/2/3tru_R activated by acupuncture (Figure 7D). ROI pairs in the contralesional hemisphere negatively correlated with each other. There were several positive pairs within the ipsilesional hemisphere and between the bilateral hemispheres (tongue/larynx sensorimotor area with the ipsilesional motor area and contralesional sensory area; Figure 7F). Group C2 patients had the activation at regions A4tl_L and A6m_R (Figure 7E). They showed numerous negative correlations over the ROI pairs. The majority of the correlations existed between the upper and lower body regions within the ipsilesional hemisphere and between the hemispheres. A few positive connections were observed between bilateral upper limb motor regions (A4ul_L and A4ul_R) and between A4ul_L, A4ul_R, and A4tl_L, and other regions (Figure 7G). Group C3 presented no significant ISC and much fewer significant ISFCs than the other two groups (Figure 7H). Positive correlations existed between ipsilesional lower limb and tongue and larynx, A6m_R and A1/2/3ll_L, and A6m_R and A4tl_R. Negative correlations existed mostly between the ipsilesional upper limb sensory area (A1/2/3ulhf_R) and contralesional cortices. Comparison among the subgroups demonstrated that stronger correlations existed in group C1 vs. group C2 (Figure 7I; p < 0.05, FDR-corrected). In conclusion, patients obtained strong and various stimulation by acupuncture at GB34 within 0–0.5 and 0.5–1 month, while fewer responses were activated by acupuncture after 1 month.

**Figure 8:**
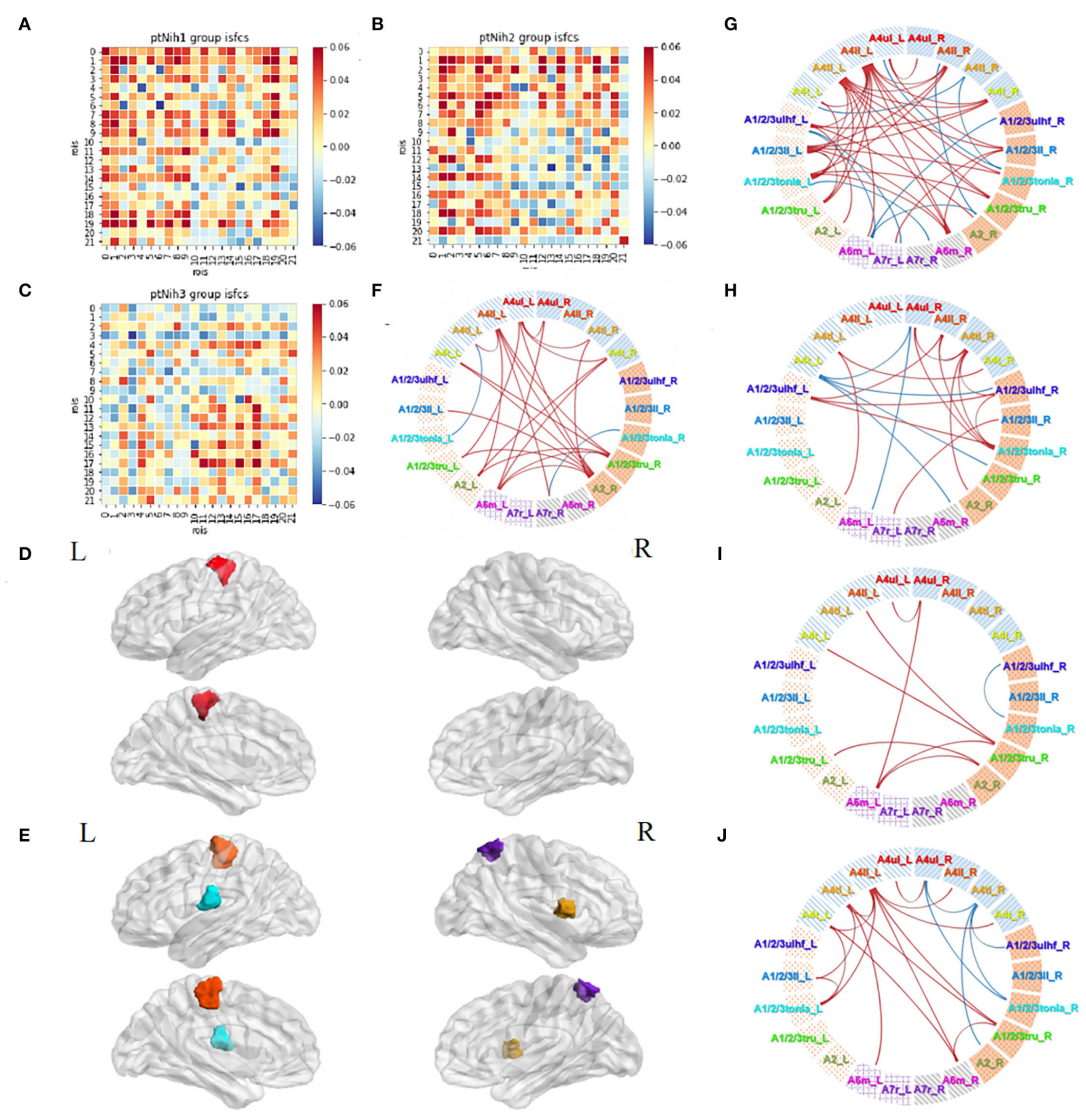
Acupuncture task-stimulated ISC and ISFC in subgroup Nihs. **(A–C)** ISFC matrix of 22 ROIs in Nih1, Nih2, and Nih3. **(D,E)** Significantly stimulated ROI in Nih1, Nih2 (*p* < 0.05, FDR-corrected). **(F–H)** Significant ISFCs in Nih1, Nih2, Nih3 group (*p* < 0.05, FDR-corrected). **(I)** Significant ISFCs between Nih1 vs. Nih3 (*p* < 0.05, FDR-corrected). **(J)** Significant ISFCs between Nih2 vs. Nih3 (*p* < 0.05, FDR-corrected).

Then, we divided the patients into three subgroups based on NIHSS severity: group Nih1 with NIHSS score of 0–1 (nearly no neural dysfunction, 18 subjects); group Nih2 with NIHSS score of 2–4 (mild dysfunction, 21 subjects); and group Nih3 with NIHSS score of 5–15 (moderate dysfunction, 24 subjects). The number and degree of the positive ISFCs decreased from the group Nih1 to Nih3 within the contralesional hemisphere and bilateral hemispheres and increased within the ipsilesional side (Figures 8A–C). Statistical analysis (*p* < 0.05, FDR-corrected) showed that the upper limb motor cortex on the unaffected side (A4ul_L) was significantly activated across the subjects in the group Nih1 (Figure 8D). Significant positive functional connectivities were mainly within bilateral upper/lower limb motor, trunk sensory, and holistic sensory regions (Figure 8F). A4ll_L, A1/2/3tonIa_L, A4tl_R, and A7r_R were activated by acupuncture in the group Nih2 (Figure 8E). The numerous significant positive connections mainly occurred within the unaffected side and between the sides. Negative correlations were mostly seen between the hemispheres (Figure 8G). In contrast, the group Nih3 showed only a few significant ISFCs involving the tongue and larynx area and the affected upper/lower limb motor area. Positive values mainly existed within the ipsilesional side (Figure 8H). Group comparison found numerous higher correlations in the contralesional side in the Nih1 group vs. the Nih3 group (Figure 8I) and between the hemispheres in the Nih2 group vs. the Nih3 group (Figure 8J). These results indicated that more and stronger isochronous connections existed in patients with lighter neural damage during acupuncture.

## Discussion

### ISC and ISFC Analyses

The ISC/ISFC analysis is a promising approach. Unlike GLM, which requires setting up *a priori* hypothetical model, the ISC/ISFC analysis provides a data-driven solution. It filters out intrinsic correlations and noise, revealing the mechanisms of brain effects induced by the task (34). Currently, this method is mostly applied to analyze fMRI data during complex stimulation or tasks. It has been proved to have less noise by previous studies (24). Current studies on the neural mechanisms of acupuncture include the effects of different experimental designs, acupuncture manipulation modality, and acupoint specificity in healthy individuals or patients. Due to the different limitations of the studies, results are often heterogeneous (35). The ISC/ISFC method, by linking the individual's brain activity to group-level activities, weakens the specificity of subjects and focuses more on the common changes induced by stimuli or tasks, making the results relatively reliable. In this study, the ISC/ISFC method was applied to describe the impaired functional activations in patients with stroke modulated by acupuncture for the first time.

### Patient Specificity

We observed huge differences in sensorimotor cortex neuroactivities during 3 fMRI runs in patients with stroke vs. normal controls. After cerebral infarction at the motor pathway, the inhibition and activation of the affected hemisphere and motor-related brain areas showed a pathological process of unilateral instability and bilateral compensation. Compared to the negative functional connectivity status at resting state in healthy subjects, patients with stroke presented positive ISFC across all ROIs, especially within the unaffected hemisphere. Research has shown that the left passive motor task activated the healthy subject's right primary sensory cortex, primary motor cortex, and somatosensory motor area (36). Based on the BN template, we described the activated regions more precisely with similar results in the control group. However, patients had a wider range of responses than normal controls. Additionally, the negative functional connectivity between the contralesional upper and lower limb motor cortex and other regions disappeared in patients compared with normal controls. Previous studies have shown that ipsilesional corticomotor excitability was initially suppressed but increased over time, brain area activation in the affected hemisphere was reduced, and activation in the unaffected hemisphere was increased after cerebral infarction (37). The impairment caused contralateral motor impairment and inter-hemispheric imbalance due to the contralesional hemisphere hyperexcitability (38). Our results agreed with this research in that the infarction increased the excitability in the contralesional motor cortices and disabled the suppression effects between the bilateral cortices. After needling at GB34, we observed broad correlation changes and significant activation at the bilateral tong/larynx area but no significant increased or decreased functional connectivity in the healthy group. A previous study has found that acupuncture at GB34 in healthy volunteers enhanced regional homogeneity (ReHo) in the right inferior parietal lobule and right frontal gyrus and reduced ReHo in the left inferior frontal gyrus (39). Our results showed trends of intersubject activation and suppression among the bilateral sensorimotor cortices stimulated by acupuncture; however, no significant between-region effects were detected. In patients with stroke, acupuncture significantly increased and decreased the ISFCs within the bilateral sensory areas, and between sensory areas and movement-imagination areas, respectively. The results in the patients agreed with previous research. The acupuncture stimulus may have individual irritants in healthy subjects rather than the common regulating effects we observed in hemiplegic patients with stroke.

### Acupuncture Effect

We designed the motor task as a reference task to explore the similar and diverse neural response patterns stimulated by acupuncture across patients or normal controls. Acupuncture triggered unique responses in the sensorimotor cortex in post-stroke hemiplegia patients. Our results showed that functional connectivities between regions A1/2/3ulhf_L and A1/2/3ll_R, A1/2/3ll_L and A6m_R increased, and A4ul_L and A1/2/3tonIa_L, A1/2/3ulhf_L and A7r_L, A7r_R and A1/2/3tonIa_L, A7r_R and A1/2/3ulhf_R decreased during the acupuncture stimulation. Previous studies of conventional FC analyses have proven that motor disorders were associated with functional connectivity abnormalities in the sensorimotor cortex in patients with stroke (40). Additionally, studies have shown that acupuncture on one side could stimulate bilateral regions (41) and increase the sensorimotor network functional connectivity in humans (42) and rats (43). Research has also shown that acupuncture increases the pain-related connectivity in the sensorimotor network after ischemic stroke or inhibits neural inflammation in the sensorimotor cortex (44, 45). A meta-analysis collected by the BN research team (29) has demonstrated that regions A1/2/3tonIa_L and A7r_L/A7r_R were involved in pain monitoring/discrimination and spatial/location discrimination, respectively. Our results indicated that the mechanism of acupuncture at GB34 in patients with stroke might include sensorimotor cortices and probably act through pain and discrimination-related responses.

Few studies have set a motor task as control of the acupuncture task. However, in Traditional Chinese Medicine theory, both procedures were used to regulate “qi” and “blood” circulation, contracting or relaxing muscles and meridians. We discovered wide-range distributions of positive ISFCs among the ROIs in the motor task vs. acupuncture. Yet, no negative values existed in the comparison map. Acupuncture may produce slighter upregulated but even downregulated stimulation compared with motor rehabilitation methods.

### Patients' Subgroup Acupuncture Effect

Subgroup calculations presented diverse features of acupuncture stimulus. Patients at the early stage of recovery (within 0.5 month) presented activation mainly in the ipsilesional motor cortex (A4ul_R) and bilateral tongue/larynx motor regions, along with negative correlations within the contralesional hemisphere. At this stage, acupuncture mainly activated the functional correlations within the affected side and between the bilateral sides (the C1 group vs. the C2 group showed positive ISFCs). From 0.5–1 month, patients developed activation in A4tl_L and A6m_R. Noticeably, patients generated significant positive correlations between the bilateral upper limb motor cortices (A4ul_R and A4ul_L) and many other negative connections between the bilateral hemispheres. Acupuncture precisely activated the bilateral upper limb regions but suppressed the widespread overreacted connections within and across the hemisphere. As the course prolonged (1–3 months), negative connections evidently reduced, with several bilateral connections between the upper body sensory cortex and other regions and a small number of positive correlations. Patients at this stage tend to have relatively smooth conditions and slow motor improvements. The acupuncture effects became personalized and lost their track from the ISFC map.

Neuroplasticity plays a crucial role during the recovery process of patients with stroke. Reorganization may be the principal process responsible for post-stroke recovery. Previous research has shown that ipsilesional corticomotor excitability was initially suppressed and increased over time in patients with stroke (37). Longitudinal studies have shown that the resting-state functional connectivity between the bilateral sensorimotor cortices decreased in the early stage and then increased in a few weeks or months during the motor recovery in patients with stroke (46, 47). In studies that performed seed-based analyses on the primary motor cortex (M1) area, the results have revealed that acupuncture increased functional connectivity between the left M1 and right M1 along with the premotor cortex (PMC), supplementary motor area (SMA), thalamus, and cerebellum (48, 49). Several Chinese clinical trials have demonstrated that patients tended to receive better motor recovery with earlier involvement in acupuncture therapy [including GB34; (50–52)]. However, no study has focused on the immediate effects of acupuncture in different time courses of ischemic stroke. Our results agreed with previous findings and identified more patterns than before. The distinct responses we observed in the subgroup Cs may be related to the pathological features of the sensorimotor cortex during motor recovery.

Visualizing the data from another angle by the severity of neural dysfunction, we found a majority of intersubject positive correlations among all three subgroups. Patients with nearly no functional damage tended to have less increased and decreased connections within the contralesional hemisphere and between the bilateral hemispheres, while patients with mild functional damage (NIHSS 2–4) had the most and strongest activation within the contralesional hemisphere and between the bilateral hemispheres. The focal regions, the contralesional upper and lower limb cortex (A4ul_L and A4ll_L) were respectively activated across the patients in the subgroups Nih1 and Nih2. Surprisingly, patients with moderate neural damage (NIHSS 5–15) activated fewer connections by acupuncture. Acupuncture generates fewer effects in the sensorimotor cortex under serious conditions.

Changes in connectivity of brain regions and between the bilateral primary sensory cortices have been significantly correlated with changes in NIHSS (53, 54). One study (55) has demonstrated that patients with an Action Research Arm Test score of 29–57 (mildly to moderately affected) presented lower connectivities in the sensorimotor network than patients with a score of 0–28 (severely affected), indicating that patients with stroke had different resting-state connectivity patterns according to severity levels. One clinical trial has found that after 10 days of acupuncture therapy, patients in the mild to moderate motor deficit group presented significantly increased functional connectivity between bilateral M1s, while the severe group had no significant change between bilateral M1s (56). Another research (57) that has focused on the immediate effects of acupuncture on the affected side-GB34 found a different enhanced and reduced response pattern of connectivity between bilateral M1, dorsal premotor cortex (PMd), and ventral premotor cortex (PMv) between the mild to moderate and severe motor deficit groups. Our findings of acupuncture effects in different subgroups by NIHSS scores may be accompanied by the characteristics of neuroplasticity (58).

### Limitations

In this study, we observed the immediate acupuncture effect at one acupoint, GB34. Although GB34 is an important acupoint in Traditional Chinese Medicine theory and alternative therapy for motor dysfunction treatment, studies have testified different effects caused by diverse acupoints. Our study provided a perspective to explore the complex mechanisms of acupuncture therapy.

We have noticed that both subgroup analyses showed distinct results from the whole patient group analysis. One reason that may explain this difference is that some of the acupuncture effects were hidden during the ISFC calculation in the whole patient group. With more homogeneity background (similar stroke time or neural damage), the patients tend to have more common reacting models to acupuncture. Another reason could be the narrowed sample size of the subgroups. One publication (59) has suggested that with 20 subjects, on average, the ISC statistics had converged close to a large sample ISC statistics with 130 subjects, however, in healthy subjects. This was the first-time application of ISFC analysis in patients with stroke. Thus, we were unsure about the reliable sample size. There should be more influence factors to consider in the further exploration.

## Conclusion

We innovatively applied ISFC approach to analyze the immediate effects of acupuncture at GB34 in sensorimotor-related cortices in post-stroke patients with motor dysfunction and normal controls. The ISFC analysis could filter out noise during the resting state. We observed special correlation patterns in patients apart from normal controls in all functional runs. Compared with the resting state and motor task results, we found that the acupuncture task triggered ISFC among the upper limb motor region, upper limb/hand/face, lower limb, and tongue/larynx sensory regions, and movement imagination regions in the patient group. The subgroup ISC and ISFC analyses of patients' acupuncture tasks showed how acupuncture stimulation changed according to disease progression and condition. Patients tended to have increased responses in the early stage of stroke (within 1 month) and decreasing responses afterward (1–3 months). Patients with mild clinical functional damage (NIHSS 2–4) tended to generate more responses *via* acupuncture than those with moderate damage (NIHSS 5–15). Our findings may help understand the clinical effects and modulatory features of acupuncture based on group-level post-stoke neuroplasticity.

The challenge we are facing is to learn more about the mechanisms of plasticity and the characteristics of acupuncture to be able to modulate them to obtain the best rehabilitation efficacy for patients with post-stroke hemiplegia. In the future, such analyses of cross-subject studies could help to optimize acupuncture regimens based on the common features under particular patients' conditions, thereby ensuring the expectations based on the possible response and outcome.

## Data Availability Statement

The raw data supporting the conclusions of this article will be made available by the authors, without undue reservation.

## Ethics Statement

The studies involving human participants were reviewed and approved by the Ethics Committee of Dongzhimen Hospital, Beijing University of Chinese Medicine. The patients/participants provided their written informed consent to participate in this study.

## Author Contributions

YuW and LW were involved in literature search, data analyses, and writing of the manuscript. YaW and ML contributed to the experimental design. LX and RL contributed to the subjects' recruitment. JWe and JWa were involved in clinical diagnosis for patients with stroke and NIHSS evaluations. HZ designed the study approaches and consulted through the study. YZ designed the study protocol and sought funding. All authors read and approved the final manuscript.

## Funding

This work was supported by grants from the National Natural Science Foundation of China (Nos. 81873257 and 81473667).

## Conflict of Interest

The authors declare that the research was conducted in the absence of any commercial or financial relationships that could be construed as a potential conflict of interest.

## Publisher's Note

All claims expressed in this article are solely those of the authors and do not necessarily represent those of their affiliated organizations, or those of the publisher, the editors and the reviewers. Any product that may be evaluated in this article, or claim that may be made by its manufacturer, is not guaranteed or endorsed by the publisher.
